# How to ‘do’ CPD with your team (from the organisation's perspective)

**Published:** 2017-05-12

**Authors:** Dhivya Ramasamy, Suzanne S. Gilbert

**Affiliations:** 1 Senior faculty Seva Foundation; 2 Senior Director, Innovation & Sight Program. Seva Foundation 1786 Fifth Street Berkeley, California, USA.

**CPD is not only the individual's responsibility but that of the organisation too. A planned programme, well designed and interactive, with an assessment element, will help avoid the pitfalls and motivate and encourage the team.**

**Figure:**
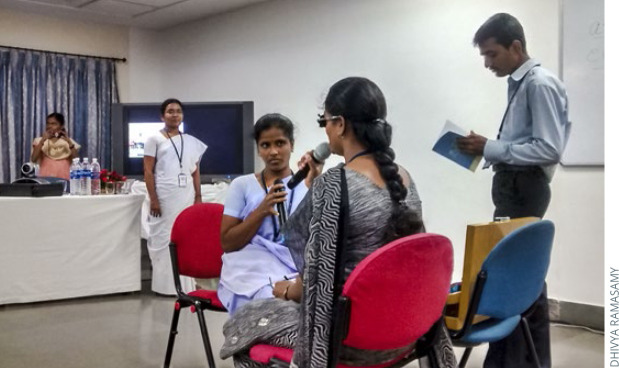
Using ‘role play’ to teach patient communication. INDIA

## How and where to start

Organisations appreciate the value of Continuing Professional Development. However, most organisations tend to be too “busy” to ensure that it happens systematically. It is often helpful for organisations to have CPD sessions planned and scheduled – say, during a given month every year. This offers regularity and helps the team to anticipate and prepare for it.

When organisations prepare for their CPD sessions, topics should be chosen with a purpose. Often, when arbitrary themes for CPD are selected – it runs the risk of being uninteresting or irrelevant to the team. CPD can be a powerful stepping stone towards organisational improvement and selection of topics can be identified through an ongoing process of continuous improvement or emerge from a systematic analysis of patient safety incident reports, patient satisfaction reports, and other information collected on an ongoing basis.

Topics can also be selected based on new technology, methods or products that are being introduced into the organisation. Reassessment of individual skills can help to identify gaps that could be addressed through retraining. Make sure that the topic you choose is in alignment with your overall institutional goals.

As CPD sessions are being planned, it is good to pin down responsibilities for different aspects of the process:

- training needs analysis
- communication
- participation
- training delivery and follow-up

It is often useful to include a representative of the audience as a CPD committee member to ensure relevance. This helps to build champions not only for the training, but for the resulting improvement process itself.

## Essentials

Ideally all CPD should be developed using good training design principles. Learning objectives should be laid down and clearly accounted for by defining the overall outcomes expected. It is essential to have a clear and focussed goal for the CPD session.

CPD sessions should avoid using old-fashioned didactic lecture format and be designed to be more engaging and interactive. The CPD audience comes with good baseline knowledge and rich experience from the field work. They can be used as a resource in the classroom by facilitating learning from each other. Performance data and real stories from within the organisation are a great resource to make the learning relevant. They also serve to emphasise the problem and highlight the opportunity to improve.

It is important to ensure that the training session includes an assessment element: e.g. a quiz or short test to gauge learning and reinforce the lesson. There are a wide array of interactive group techniques, including role playing and small group demonstrations that can reveal the extent to which learners have learned. This assures that the CPD is accountable and is grounded upon expected outcomes.

## Common pitfalls

**Lack of institutional “buy-in”:** CPD activities can be ineffective, especially when they are deployed as a standalone event without a champion among the organisation's leadership. The audience must be able to perceive the CPD activity (and the overall improvement process) as a priority for the organisation and directed towards improving patient care.

**Have the right people involved:** Leaders must ensure that CPD sessions are seen as an important investment. Plan that the right staff are sent to the CPD. Where only a headcount is expected, often the most junior staff are sent to the training when they alone cannot influence the improvement process.

**Training alone cannot solve everything:** Sometimes, the best planned CPD cannot bring out the desired improvement. It is important to determine if training can create the solution or if policies, procedures, and other systems-related factors require changing.

## Addressing barriers to learning

Select appropriate teaching methods: Often, small changes to the training design can help improve learning. If you are teaching a practical skill or process, it helps to keep the session practical: give a demonstration, create an opportunity for participants to practise the skill in class and learn from each other's mistakes. Similarly, role plays and videos can help to engage the audience for “soft” themes such as patient-centred care and communication.

Maintain relevance for the audience: Trainers or faculty must have enough experience and competence on the topic and be given sufficient time to prepare for the session. It is also important for the trainers to be aware of the “level” of the learners – a pre-test often helps to do this. It also helps the audience open up to what is going to be taught. If the faculty is from outside the organisation, it helps to “orient” them to the organisation. This will help the faculty to adjust the style and language appropriate for the audience.

State expectation from the onset: Is the CPD structured to help the audience translate what is taught in their own workplace? It is important to explicitly bring out how the training programme connects to the work of each staff member and what they are expected to change or do after the CPD.

## How to encourage and motivate the team over the long term

As CPD activities are usually meant to be a part of a change or continuous improvement process, it is important to maintain the motivation of the learner, beyond the session itself. Recognition – a badge or certificate announcing that they have completed the training – can help create a sense of achievement.

It is important to have a follow-up action plan beyond the training session to ensure that the improvement process is set in motion. This follow-up action will have to be adequately resourced and reinforced regularly by leaders. Posters and flowcharts can serve as reinforcements of CPD lessons.

In the long run, it is the measurement of outcomes and the communication of progress that is important to keep the team motivated and engaged in the overall improvement process.

**Case study 1**

**Lila Puri** *Ophthalmologist, Nepal*

I took the Global Blindness course (**http://iceh.lshtm.ac.uk/oer/**) while practising as an ophthalmologist in a high volume community eye hospital in Nepal.

Our programme was already delivering many of the public health activities covered by the course but I still found it very useful for refreshing my knowledge on both the principles of public health for eye care and its application. The course was a great opportunity to hear from world experts in public health eye care – I felt as if I was in the class physically!

We were also able to share our experiences, ideas and strategies. For a short six-week course, the content was very comprehensive. I had new insights into planning and prioritising for sustainable, equitable and accessible services. This was very helpful for me as I have a decision-making position in my organisation.

Another great outcome of this course was that it motivated me to go on to formal study and apply to LSHTM where I am now studying for the Masters in Public Health Eye Care. My studies here have shown me even more how useful the Global Blindness course is as a resource for all eye care cadres.

## The improvement opportunity: enhance staff-patient communication

**Evidence used:** Patient satisfaction surveys, patient interviews, focus group discussions with patient and their families, suggestion books.

**Improvement plan:** Improve patient communication by creating awareness for importance of good patient communication and train doctors and allied health personnel on best practices for effective communication.

**CPD on Patient Communication:** A half-day workshop focused on patient communication using videos of patient interviews and patient stories to sensitise staff about patient perspectives. A qualified counsellor was invited as faculty and given patient stories to “orient” her to the problem. The audience were engaged in the learning process using stories and role-plays. Clear guidelines were laid out for patient communication.

**Outcome measures:** Percentage of “Excellent” ratings on overall satisfaction.

**EYEXCEL– Expanding the Eye Care Workforce through Excellence in Training**

July 11–14, 2017, Madurai, India

This four-day course helps eye care institutions to develop or improve their training programmes for allied ophthalmic personnel. Teams take a comprehensive look at training: training design, training administration, sharing available training resources, using technology in training, and making the training sustainable. Thus far teams from more than 100 eye hospitals have participated in Eyexcel.

Visit **www.aurovikas.co.in/WebCourseProfile.aspx?tmpcoursecode=C000000425** for more details and to register for the course.

Co-sponsored by Aravind, Seva Foundation and the International Council of Ophthalmology.

**Case study 2**

**Shalinder Sabherwal** *Ophthalmologist, India*

I studied the Global Blindness free online course (**http://iceh.lshtm.ac.uk/oer/**) while practising as an ophthalmologist in Delhi. I appreciated the flexible format which meant I could work and study at the same time. It was my first opportunity to learn about eye care planning and management. I was able to engage with global experts which helped deepen my understanding of the key issues.

Learning together with other eye care professionals from different countries, cadres and local settings exposed me to many new experiences and ideas. The Global Blindness course inspired me to think about how our free rural outreach cataract programme could address some of the patient barriers we were seeing. Around 25% of the people who were offered surgery did not arrive at the hospital afterwards. From a survey with patients we identified that patients were afraid of a poor quality surgical outcome and we were carrying out surgeries during harvest which meant there was a lack of people to escort them to hospital.

We addressed these barriers through counselling patients and their relatives about surgical quality and we re-scheduled the timing of surgeries. Taking the Global Blindness course helped me reflect on and improve our eye care outreach programme.

